# Supplementary material for: 2-[18F]FDG-PET/CT in Cancer of Unknown Primary Tumor—A Retrospective Register-Based Cohort Study
Source: J Imaging. 2023 Aug 31;9(9):178. doi: 10.3390/jimaging9090178 (PMC10532746; doi:10.3390/jimaging9090178)
Supplement: Supplementary file 1 [file jimaging-09-00178-s001.zip › ESM_1.pdf]

Title: 2-[^{18}F]FDG-PET/CT in cancer of unknown primary tumour – a retrospective register-based cohort study

Journal: Journal of Imaging

Authors: Heidi Rimer*, Melina S. Jensen*, Sara E. Dahlsgaard-Wallenius, Lise Eckhoff, Peter Thye-Rønn, Charlotte Kristiansen, Malene G. Hildebrandt, Oke Gerke

**Shared co-first authorship*

Corresponding author: Oke Gerke; University of Southern Denmark, Department of Clinical Research, Odense, Denmark and Odense University Hospital, Department of Nuclear Medicine, Odense, Denmark

Corresponding author E-mail: Oke.Gerke@rsyd.dk

Online Resource 1: Types of tissue found in metastasis biopsy

	**2-[^{18}F]FDG-PET/CT (N=159)**	**CT (N=34)**	**P-value**
Adenocarcinoma	61 (38.4%)	18 (52.9%)	0.12
Undifferentiated carcinoma	38 (23.9%)	5 (14.7%)	0.24
Undifferentiated malignant cells	27 (17.0%)	7 (20.6%)	0.62
Neuroendocrine carcinoma	11 (6.9%)	3 (8.8%)	0.70
Squamous cell carcinoma	11 (6.9%)	1 (2.9%)	0.38
Other	11 (6.9%)	0	0.11
Sarcoma	2 (1.3%)	0	0.51
Malignant melanoma	1 (0.6%)	0	0.64

Supplemental Table 1 *Types of tissue found in a metastasis biopsy, comparing CUP patients who received a 2-[^{18}F]FDG-PET/CT with patients who received a CT using a Chi-squared test. CUP: Cancer of unknown primary tumour. CT: Computed tomography. 2-[^{18}F]FDG: 2-deoxy-2-[^{18}F]fluoro-D-glucose. PET: Positron emission tomography.*
