# Supplementary material for: 2-[18F]FDG-PET/CT in Cancer of Unknown Primary Tumor—A Retrospective Register-Based Cohort Study
Source: J Imaging. 2023 Aug 31;9(9):178. doi: 10.3390/jimaging9090178 (PMC10532746; doi:10.3390/jimaging9090178)
Supplement: Supplementary file 1 [file jimaging-09-00178-s001.zip › ESM_2.pdf]

Title: 2-[^{18}F]FDG-PET/CT in cancer of unknown primary tumour – a retrospective register-based cohort study

Journal: Journal of Imaging

Authors: Heidi Rimer*, Melina S. Jensen*, Sara E. Dahlsgaard-Wallenius, Lise Eckhoff, Peter Thye-Rønn, Charlotte Kristiansen, Malene G. Hildebrandt, Oke Gerke

**Shared co-first authorship*

Corresponding author: Oke Gerke; University of Southern Denmark, Department of Clinical Research, Odense, Denmark and Odense University Hospital, Department of Nuclear Medicine, Odense, Denmark

Corresponding author E-mail: Oke.Gerke@rsyd.dk

Online Resource 2: Propensity score analysis

N=193	Coefficient (95% CI)	P-value
Sex<br>- Male	 -0.34 (-0.80-0.12)	 0.15
Age at first hospital visit	0.0024 (-0.018-0.023)	0.82
Location of first hospital visit	-0.0038 (-0.012-0.0039)	0.33
Previously known cancer diagnoses<br>- 1<br>- 2 or >2	 0.59 (-0.041-1.23)<br>-1.05 (-2.16-0.052)	 0.067<br>0.062
Performance status<br>- 1<br>- 2<br>- 3<br>- 4<br>- Not obtained	 -0.47 (-1.33-0.38)<br>-0.37 (-1.41-0.67)<br>-1.32 (-2.21-(-0.42))<br>-1.12 (-2.35-0.098)<br>-0.66 (-1.39-0.070)	 0.28<br>0.49<br>0.004<br>0.071<br>0.076
Comorbidities	-0.069 (-0.21-0.067)	0.32

Supplemental Table 2 *Factors contributing to the likelihood of CUP patients receiving a 2-[^{18}F]FDG-PET/CT. CUP: Cancer of unknown primary tumour. CT: Computed tomography. 2-[^{18}F]FDG: 2-deoxy-2-[^{18}F]fluoro-D-glucose. PET: Positron emission tomography.*

	2-[^{18}F]FDG-PET/CT (N=159)	CT (N=34)	P-value
Mean propensity score (95% CI)	0.84 (0.82-0.86)	0.74 (0.69-0.79)	<0.001

Supplemental Table 3 *Comparison of propensity scores of CUP patients who received a 2-[^{18}F]FDG-PET/CT and patients who received a CT, respectively, using an unpaired t-test. CUP: Cancer of unknown primary tumour. CT: Computed tomography. 2-[^{18}F]FDG: 2-deoxy-2-[^{18}F]fluoro-D-glucose. PET: Positron emission tomography.*

Title: 2-[18F]FDG-PET/CT in cancer of unknown primary tumour – a retrospective register-based cohort study

Journal: Journal of Imaging

Authors: Heidi Rimer*, Melina S. Jensen*, Sara E. Dahlsgaard-Wallenius, Lise Eckhoff, Peter Thye-Rønn, Charlotte Kristiansen, Malene G. Hildebrandt, Oke Gerke

Shared co-first authorship

Corresponding author: Oke Gerke; University of Southern Denmark, Department of Clinical Research, Odense, Denmark and Odense University Hospital, Department of Nuclear Medicine, Odense, Denmark

Corresponding author E-mail: Oke.Gerke@rsyd.dk

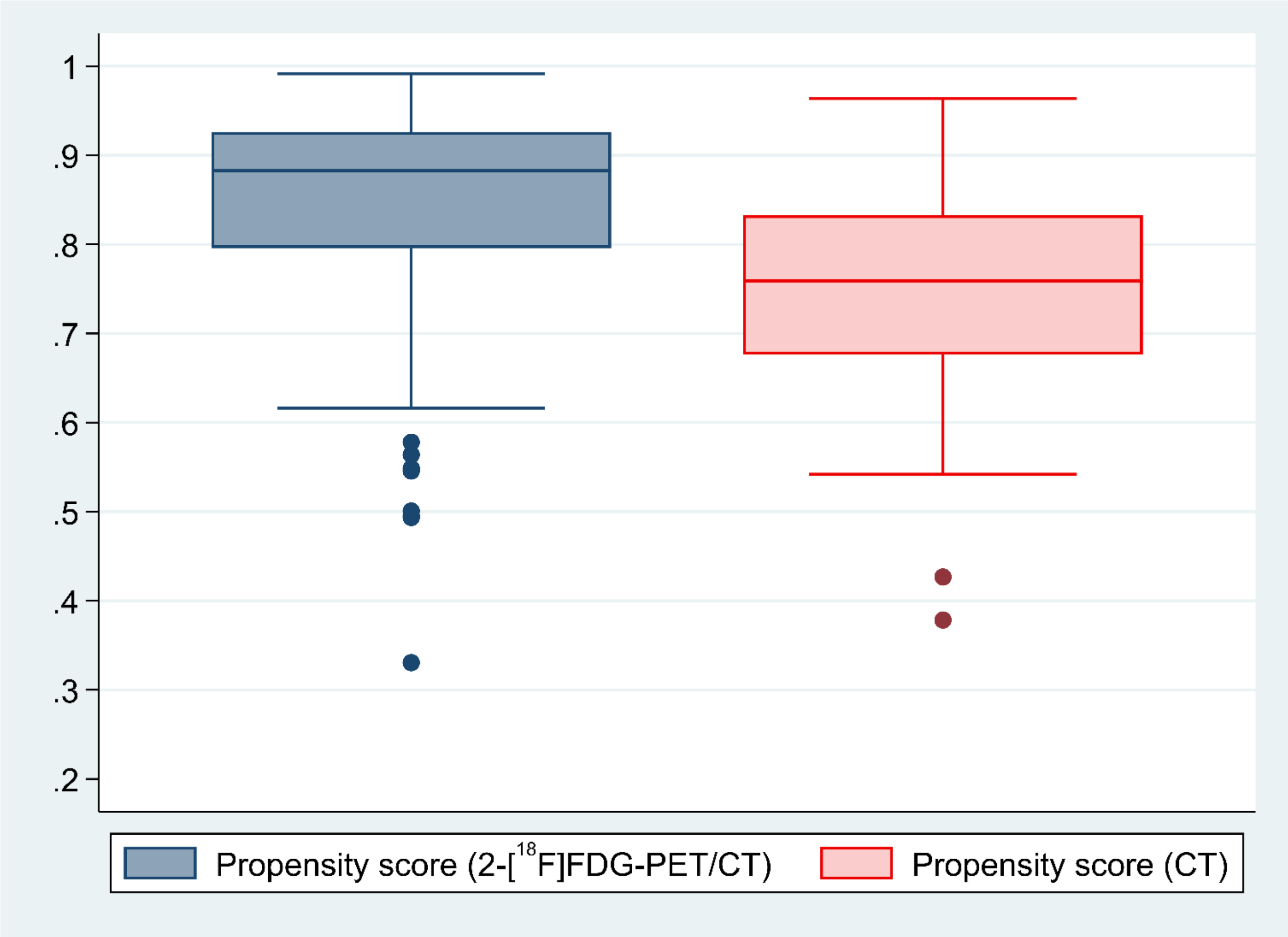

Supplemental Fig. 1 *Boxplot representing propensity scores based on background characteristics, comparing CUP patients who received a 2-[18F]FDG-PET/CT with patients who received a CT. CUP: Cancer of unknown primary tumour. CT: Computed tomography. 2-[18F]FDG: 2-deoxy-2-[18F]fluoro-D-glucose. PET: Positron emission tomography.*

For 2-[18F]FDG-PET/CT, median propensity score was 88%, while the interquartile range was 80% to 93% and minimum/maximum was 33% and 99%, respectively. For CT, median propensity score was 76%, while the interquartile range was 68% to 83% and minimum/maximum was 38% and 96%, respectively
