# Supplementary material for: 2-[18F]FDG-PET/CT in Cancer of Unknown Primary Tumor—A Retrospective Register-Based Cohort Study
Source: J Imaging. 2023 Aug 31;9(9):178. doi: 10.3390/jimaging9090178 (PMC10532746; doi:10.3390/jimaging9090178)
Supplement: Supplementary file 1 [file jimaging-09-00178-s001.zip › ESM_3.pdf]

Title: 2-[18F]FDG-PET/CT in cancer of unknown primary tumour – a retrospective register-based cohort study

Journal: Journal of Imaging

Authors: Heidi Rimer*, Melina S. Jensen*, Sara E. Dahlsgaard-Wallenius, Lise Eckhoff, Peter Thye-Rønn, Charlotte Kristiansen, Malene G. Hildebrandt, Oke Gerke

*Shared co-first authorship*

Corresponding author: Oke Gerke; University of Southern Denmark, Department of Clinical Research, Odense, Denmark and Odense University Hospital, Department of Nuclear Medicine, Odense, Denmark

Corresponding author E-mail: Oke.Gerke@rsyd.dk

## Online Resource 3: Survival (subgroup analysis on the impact of a definitive diagnosis)

| **Variable** | **Crude univariable model** | | | **Multivariable model 1: adjusted for propensity score** | | | **Multivariable model 2: adjusted for age, sex, treatment and propensity score** | | |
|---|---|---|---|---|---|---|---|---|---|
| | HR | 95% CI | P-value | HR | 95% CI | P-value | HR | 95% CI | P-value |
| PET/CT | 0.65 | 0.38-1.12 | 0.12 | 0.92 | 0.51-1.64 | 0.77 | 0.74 | 0.41-1.36 | 0.33 |
| Age | | | | | | | 1.01 | 1.00-1.03 | 0.13 |
| Sex (Male) | | | | | | | 1.06 | 0.71-1.60 | 0.78 |
| Treatment (yes) | | | | | | | 0.34 | 0.21-0.54 | <0.001 |
| Treatment (unknown) | | | | | | | 0.40 | 0.05-3.06 | 0.38 |
| Propensity score | | | | 0.11 | 0.03-0.46 | 0.003 | 0.32 | 0.06-1.69 | 0.18 |

***Supplemental Table 4*** *Cox-regression regarding survival in a subgroup of CUP patients who received a definitive diagnosis, presenting the hazard ratio of patients who received a 2-[18F]FDG-PET/CT with patients who received a CT as baseline. CUP: Cancer of unknown primary tumour. CT: Computed tomography. 2-[18F]FDG: 2-deoxy-2-[18F]fluoro-D-glucose. PET: Positron emission tomography.*

Title: 2-[18F]FDG-PET/CT in cancer of unknown primary tumour – a retrospective register-based cohort study

Journal: Journal of Imaging

Authors: Heidi Rimer*, Melina S. Jensen*, Sara E. Dahlsgaard-Wallenius, Lise Eckhoff, Peter Thye-Rønn, Charlotte Kristiansen, Malene G. Hildebrandt, Oke Gerke

*Shared co-first authorship*

Corresponding author: Oke Gerke; University of Southern Denmark, Department of Clinical Research, Odense, Denmark and Odense University Hospital, Department of Nuclear Medicine, Odense, Denmark

Corresponding author E-mail: Oke.Gerke@rsyd.dk

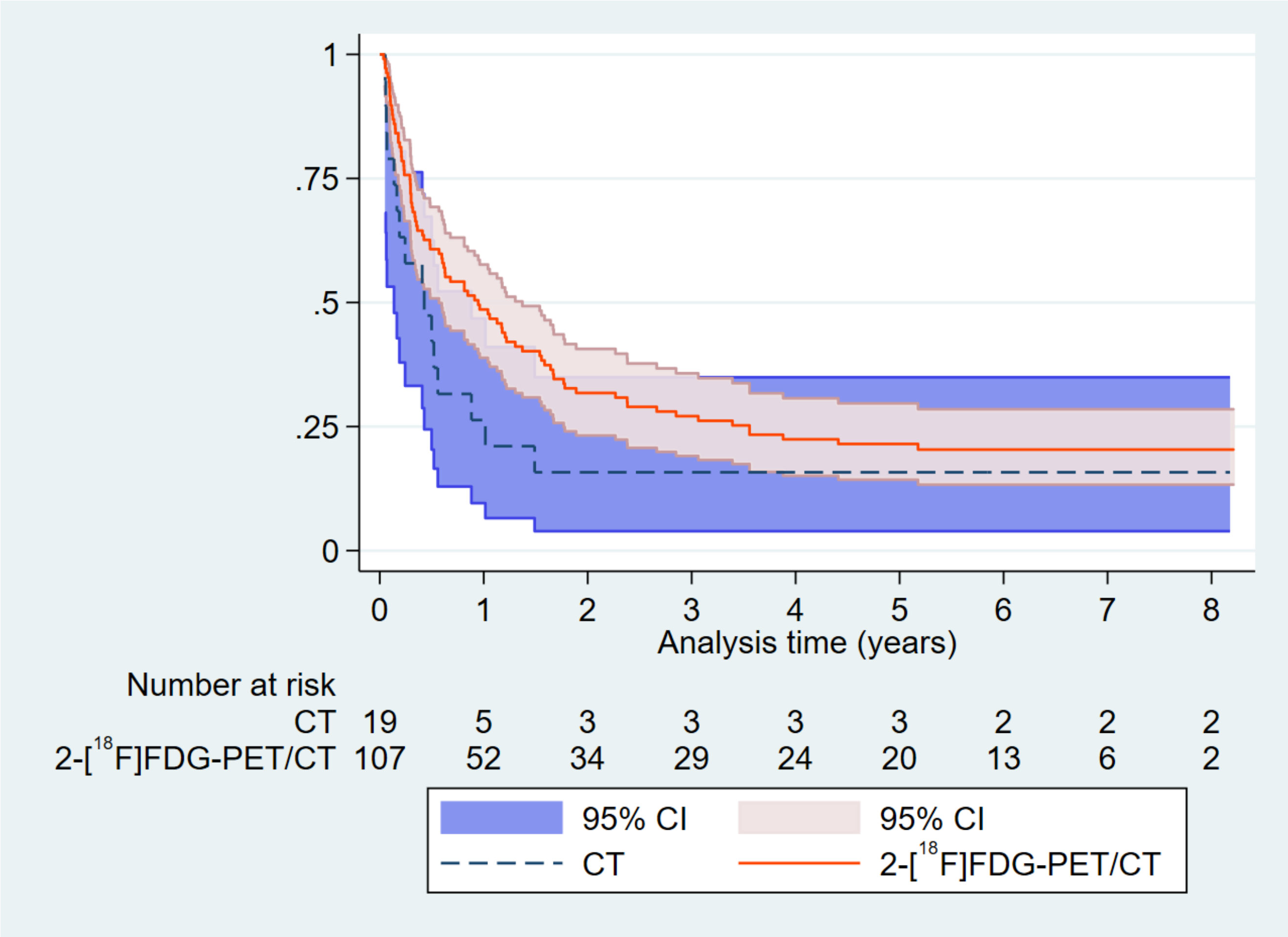


***Supplemental Fig. 2*** *Kaplan-Meier survival estimates in a subgroup of CUP patients who received a final diagnosis, comparing patients who received a 2-[18F]FDG-PET/CT with patients who received a CT. CUP: Cancer of unknown primary tumour. CT: Computed tomography. 2-[18F]FDG: 2-deoxy-2-[18F]fluoro-D-glucose. PET: Positron emission tomography.*

*The number of patients at risk decreased from 19 to 5 in the CT group and from 107 to 52 in the 2-[18F]FDG-PET/CT group within one year. Within two years, this number further decreased to 3 in the CT group and 34 in the 2-[18F]FDG-PET/CT group, while the numbers after five years were 3 in the CT group and 20 in the 2-[18F]FDG-PET/CT group, respectively. Although survival in the 2-[18F]FDG-PET/CT group was consistently higher than in the CT group, none of this proved statistically significant due to overlapping 95% confidence intervals*

Title: 2-[$^{18}$F]FDG-PET/CT in cancer of unknown primary tumour – a retrospective register-based cohort study

Journal: Journal of Imaging

Authors: Heidi Rimer*, Melina S. Jensen*, Sara E. Dahlsgaard-Wallenius, Lise Eckhoff, Peter Thye-Rønn, Charlotte Kristiansen, Malene G. Hildebrandt, Oke Gerke

*Shared co-first authorship*

Corresponding author: Oke Gerke; University of Southern Denmark, Department of Clinical Research, Odense, Denmark and Odense University Hospital, Department of Nuclear Medicine, Odense, Denmark

Corresponding author E-mail: Oke.Gerke@rsyd.dk

| **Variable** | **Crude univariable model** | | | **Multivariable model 1: adjusted for propensity score** | | | **Multivariable model 2: adjusted for age, sex, treatment and propensity score** | | |
|---|---|---|---|---|---|---|---|---|---|
| | HR | 95% CI | P-value | HR | 95% CI | P-value | HR | 95% CI | P-value |
| PET/CT | 0.67 | 0.37-1.22 | 0.19 | 0.80 | 0.43-1.46 | 0.46 | 0.61 | 0.32-1.20 | 0.13 |
| Age | | | | | | | 1.02 | 0.99-1.05 | 0.18 |
| Sex (Male) | | | | | | | 1.68 | 0.89-3.18 | 0.11 |
| Treatment (yes) | | | | | | | 0.39 | 0.20-0.74 | 0.004 |
| Treatment (unknown) | | | | | | | <0.001 | N/A | 1.00 |
| Propensity score | | | | 0.06 | 0.01-0.57 | 0.015 | 0.16 | 0.01-1.79 | 0.14 |

***Supplemental Table 5*** *Cox-regression regarding survival in a subgroup of CUP patients who did not receive a definitive diagnosis, presenting the hazard ratio of patients who received a 2-[$^{18}$F]FDG-PET/CT with patients who received a CT as baseline. CUP: Cancer of unknown primary tumour. CT: Computed tomography. 2-[$^{18}$F]FDG: 2-deoxy-2-[$^{18}$F]fluoro-D-glucose. PET: Positron emission tomography.*

Title: 2-[$^{18}$F]FDG-PET/CT in cancer of unknown primary tumour – a retrospective register-based cohort study

Journal: Journal of Imaging

Authors: Heidi Rimer*, Melina S. Jensen*, Sara E. Dahlsgaard-Wallenius, Lise Eckhoff, Peter Thye-Rønn, Charlotte Kristiansen, Malene G. Hildebrandt, Oke Gerke

*Shared co-first authorship*

Corresponding author: Oke Gerke; University of Southern Denmark, Department of Clinical Research, Odense, Denmark and Odense University Hospital, Department of Nuclear Medicine, Odense, Denmark

Corresponding author E-mail: Oke.Gerke@rsyd.dk

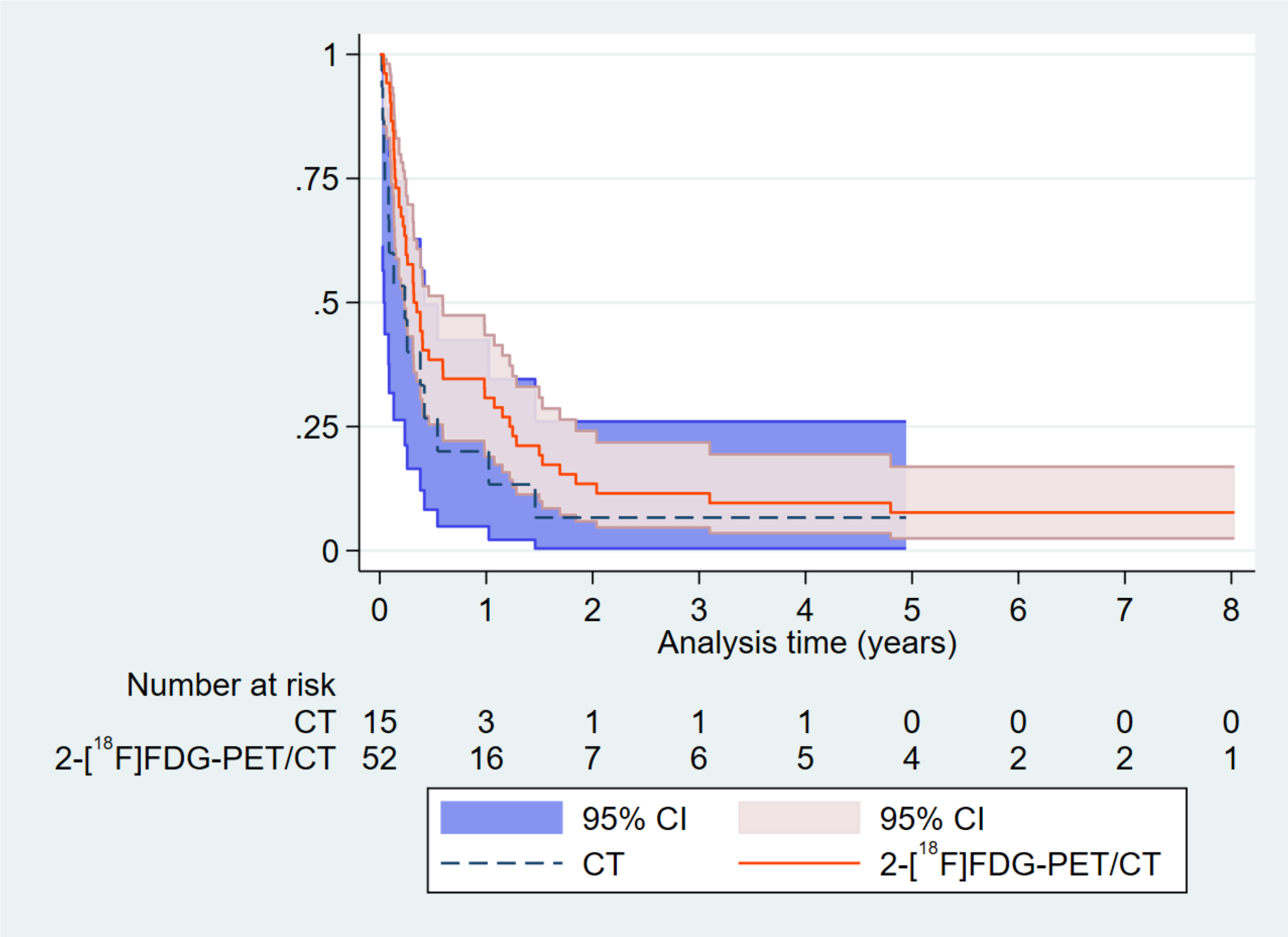


***Supplemental Fig. 3*** *Kaplan-Meier survival estimates in a subgroup of CUP patients who did not receive a final diagnosis, comparing patients who received a 2-[$^{18}$F]FDG-PET/CT with patients who received a CT. CUP: Cancer of unknown primary tumour. CT: Computed tomography. 2-[$^{18}$F]FDG: 2-deoxy-2-[$^{18}$F]fluoro-D-glucose. PET: Positron emission tomography.*

*The number of patients at risk decreased from 15 to 3 in the CT group and from 52 to 16 in the 2-[$^{18}$F]FDG-PET/CT group within one year. Within two years, this number further decreased to 1 in the CT group and 7 in the 2-[$^{18}$F]FDG-PET/CT group, while the numbers after five years were 0 in the CT group and 4 in the 2-[$^{18}$F]FDG-PET/CT group, respectively. Although survival in the 2-[$^{18}$F]FDG-PET/CT group was consistently higher than in the CT group, none of this proved statistically significant due to overlapping 95% confidence intervals*
