# Supplementary material for: 2-[18F]FDG-PET/CT in Cancer of Unknown Primary Tumor—A Retrospective Register-Based Cohort Study
Source: J Imaging. 2023 Aug 31;9(9):178. doi: 10.3390/jimaging9090178 (PMC10532746; doi:10.3390/jimaging9090178)
Supplement: Supplementary file 1 [file jimaging-09-00178-s001.zip › ESM_4.pdf]

Title: 2-[18F]FDG-PET/CT in cancer of unknown primary tumour – a retrospective register-based cohort study

Journal: Journal of Imaging

Authors: Heidi Rimer*, Melina S. Jensen*, Sara E. Dahlsgaard-Wallenius, Lise Eckhoff, Peter Thye-Rønn, Charlotte Kristiansen, Malene G. Hildebrandt, Oke Gerke

**Shared co-first authorship*

Corresponding author: Oke Gerke; University of Southern Denmark, Department of Clinical Research, Odense, Denmark and Odense University Hospital, Department of Nuclear Medicine, Odense, Denmark

Corresponding author E-mail: Oke.Gerke@rsyd.dk

Online Resource 4: Time to final diagnosis

**Group**	**CT**	**2-[18F]FDG-PET/CT**	**Total**
Number of patients	34	159	193
- Number of patients with definitive diagnosis	19 (55.9%)	107 (67.3%)	126 (65.3%)
Total follow-up time (days)	3030	9770	12800
- Patients with definitive diagnosis	803	6326	7129
Median (min-max) time to final diagnosis (days)	21 (7-1806)	35 (5-619)	33 (5-1806)
- Patients with definitive diagnosis	21 (7-250)	33 (5-484)	31 (5-484)

Supplemental Table 6 *Overview on time to final diagnosis amongst CUP patients who received a 2-[18F]FDG-PET/CT scan or a CT scan, respectively. CUP: Cancer of unknown primary tumour. CT: Computed tomography. 2-[18F]FDG: 2-deoxy-2-[18F]fluoro-D-glucose. PET: Positron emission tomography.*

**Variable**	**Crude univariable model**			**Multivariable model 1: adjusted for propensity-score**			**Multivariable model 2: adjusted for age, sex and propensity score**		
	HR	95% CI	P-value	HR	95% CI	P-value	HR	95% CI	P-value
PET/CT	0.72	0.49-1.05	0.090	0.74	0.50-1.12	0.15	0.72	0.48-1.09	0.12
Age							1.00	0.99-1.01	0.89
Sex (Male)							1.30	0.97-1.74	0.082
Propensity score				0.75	0.21-2.67	0.66	0.94	0.26-3.42	0.93

Supplemental Table 7 *Cox-regression regarding time to final diagnosis in CUP patients, presenting the hazard ratio of patients who received a 2-[18F]FDG-PET/CT with patients who received a CT as baseline. CUP: Cancer of unknown primary tumour. CT: Computed tomography. 2-[18F]FDG: 2-deoxy-2-[18F]fluoro-D-glucose. PET: Positron emission tomography.*

Title: 2-[^{18}F]FDG-PET/CT in cancer of unknown primary tumour – a retrospective register-based cohort study

Journal: Journal of Imaging

Authors: Heidi Rimer*, Melina S. Jensen*, Sara E. Dahlsgaard-Wallenius, Lise Eckhoff, Peter Thye-Rønn, Charlotte Kristiansen, Malene G. Hildebrandt, Oke Gerke

Shared co-first authorship

Corresponding author: Oke Gerke; University of Southern Denmark, Department of Clinical Research, Odense, Denmark and Odense University Hospital, Department of Nuclear Medicine, Odense, Denmark

Corresponding author E-mail: Oke.Gerke@rsyd.dk

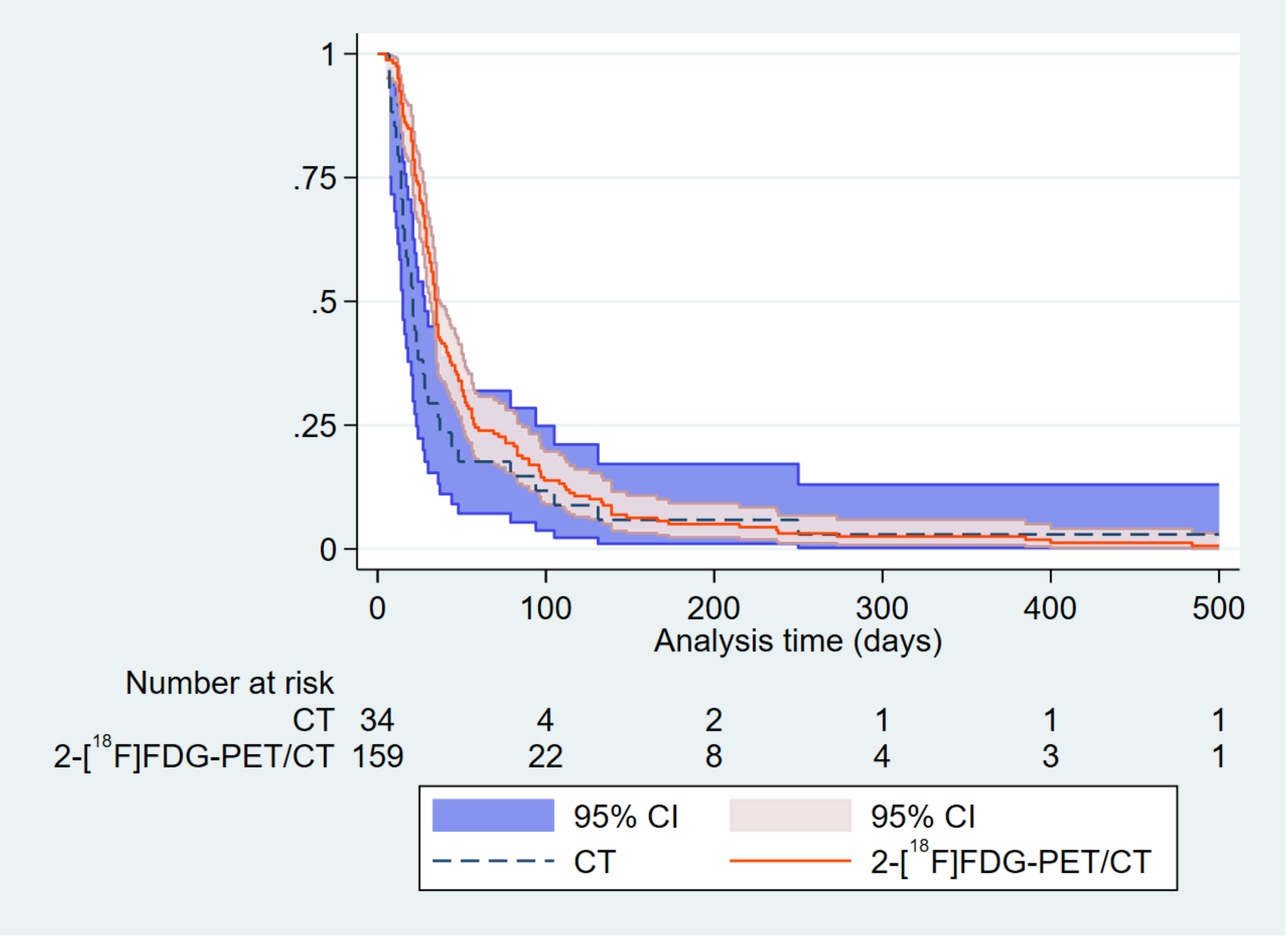

Supplemental Fig. 4 *Kaplan-Meier estimates regarding time to final diagnosis in CUP patients, comparing patients who received a 2-[^{18}F]FDG-PET/CT with patients who received a CT. CUP: Cancer of unknown primary tumour. CT: Computed tomography. 2-[^{18}F]FDG: 2-deoxy-2-[^{18}F]fluoro-D-glucose. PET: Positron emission tomography.*

The number of patients at risk decreased from 34 to 4 in the CT group and from 159 to 22 in the 2-[^{18}F]FDG-PET/CT group within 100 days. Within 200 days, this number further decreased to 2 in the CT group and 8 in the 2-[^{18}F]FDG-PET/CT group, while the numbers after 500 days were 1 in both groups. None of this proved statistically significant due to overlapping 95% confidence intervals

Title: 2-[^{18}F]FDG-PET/CT in cancer of unknown primary tumour – a retrospective register-based cohort study

Journal: Journal of Imaging

Authors: Heidi Rimer*, Melina S. Jensen*, Sara E. Dahlsgaard-Wallenius, Lise Eckhoff, Peter Thye-Rønn, Charlotte Kristiansen, Malene G. Hildebrandt, Oke Gerke

Shared co-first authorship

Corresponding author: Oke Gerke; University of Southern Denmark, Department of Clinical Research, Odense, Denmark and Odense University Hospital, Department of Nuclear Medicine, Odense, Denmark

Corresponding author E-mail: Oke.Gerke@rsyd.dk

**Variable**	**Crude univariable model**			**Multivariable model 1: adjusted for propensity-score**			**Multivariable model 2: adjusted for age, sex and propensity score**		
	HR	95% CI	P-value	HR	95% CI	P-value	HR	95% CI	P-value
PET/CT	0.66	0.40-1.08	0.098	0.69	0.40-1.20	0.18	0.65	0.37-1.13	0.13
Age							1.00	0.99-1.02	0.78
Sex (Male)							1.55	1.06-2.28	0.025
Propensity score				0.79	0.17-3.67	0.77	1.30	0.26-6.48	0.75

Supplemental Table 8 *Cox-regression regarding time to final diagnosis in a subgroup of CUP patients who specifically received a definitive diagnosis, presenting the hazard ratio of patients who received a 2-[^{18}F]FDG-PET/CT with patients who received a CT as baseline. CUP: Cancer of unknown primary tumour. CT: Computed tomography. 2-[^{18}F]FDG: 2-deoxy-2-[^{18}F]fluoro-D-glucose. PET: Positron emission tomography.*

Title: 2-[18F]FDG-PET/CT in cancer of unknown primary tumour – a retrospective register-based cohort study

Journal: Journal of Imaging

Authors: Heidi Rimer*, Melina S. Jensen*, Sara E. Dahlsgaard-Wallenius, Lise Eckhoff, Peter Thye-Rønn, Charlotte Kristiansen, Malene G. Hildebrandt, Oke Gerke

Shared co-first authorship

Corresponding author: Oke Gerke; University of Southern Denmark, Department of Clinical Research, Odense, Denmark and Odense University Hospital, Department of Nuclear Medicine, Odense, Denmark

Corresponding author E-mail: Oke.Gerke@rsyd.dk

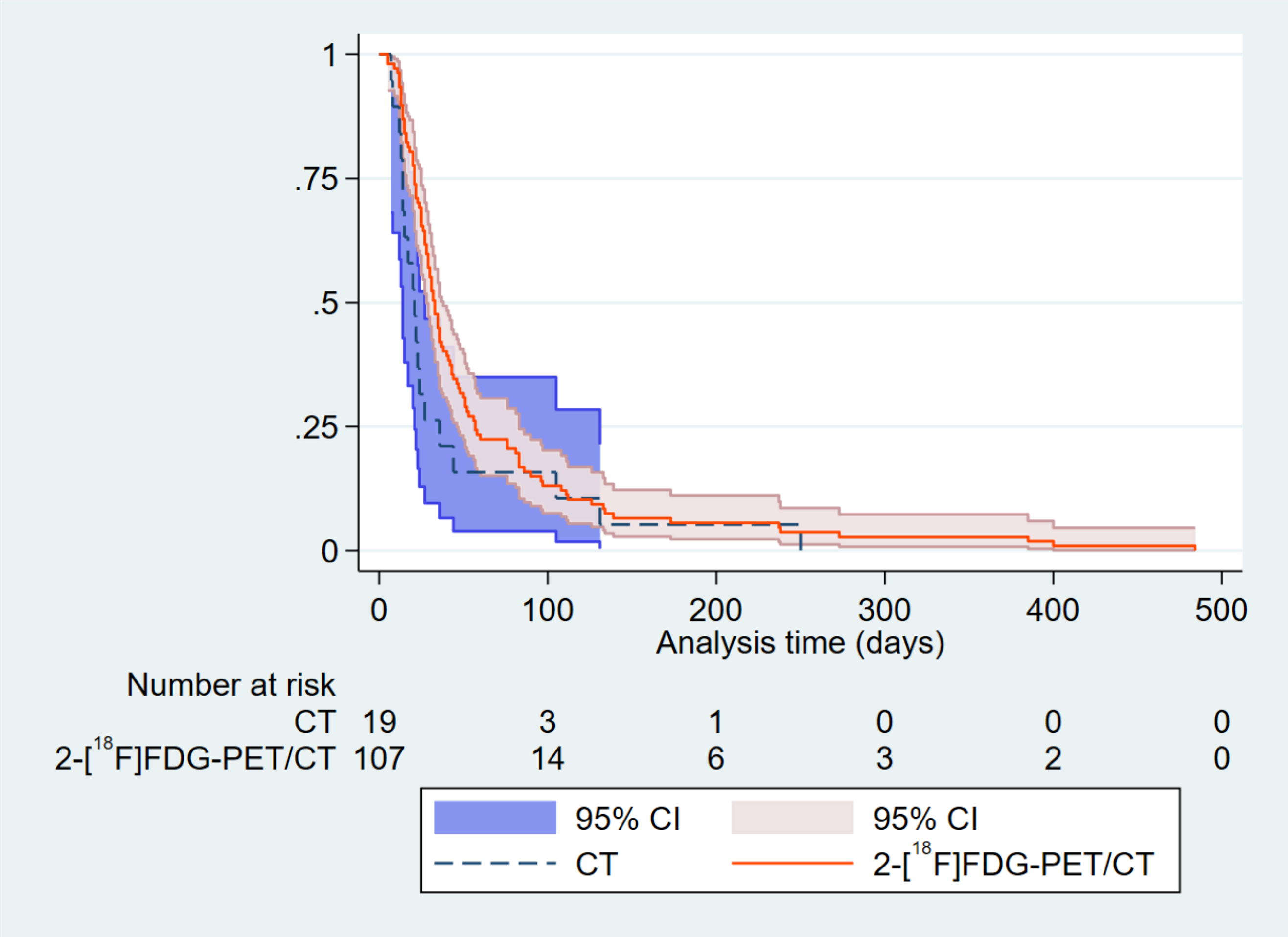

Supplemental Fig. 5 *Kaplan-Meier estimates regarding time to final diagnosis in a subgroup of CUP patients who specifically received a definitive diagnosis, comparing patients who received a 2-[18F]FDG-PET/CT with patients who received a CT. CUP: Cancer of unknown primary tumour. CT: Computed tomography. 2-[18F]FDG: 2-deoxy-2-[18F]fluoro-D-glucose. PET: Positron emission tomography.*

The number of patients at risk decreased from 19 to 3 in the CT group and from 107 to 14 in the 2-[18F]FDG-PET/CT group within 100 days. Within 200 days, this number further decreased to 1 in the CT group and 6 in the 2-[18F]FDG-PET/CT group, while the numbers after 500 days were 0 in both groups. None of this proved statistically significant due to overlapping 95% confidence intervals
